# Comparative Risks of Potential Adverse Events Following COVID-19 mRNA Vaccination Among Older US Adults

**DOI:** 10.1001/jamanetworkopen.2023.26852

**Published:** 2023-08-02

**Authors:** Daniel A. Harris, Kaleen N. Hayes, Andrew R. Zullo, Vincent Mor, Preeti Chachlani, Yalin Deng, Ellen P. McCarthy, Djeneba Audrey Djibo, Cheryl N. McMahill-Walraven, Stefan Gravenstein

**Affiliations:** 1 Center for Gerontology and Healthcare Research, Brown University School of Public Health, Providence, Rhode Island; 2 Department of Health Services, Policy, and Practice, Brown University School of Public Health, Providence, Rhode Island; 3 Department of Epidemiology, Brown University School of Public Health, Providence, Rhode Island; 4 Providence Medical Center Veterans Administration Research Service, Providence, Rhode Island; 5 Hinda and Arthur Marcus Institute for Aging Research, Hebrew SeniorLife, Boston, Massachusetts; 6 Division of Gerontology, Department of Medicine, Beth Israel Deaconess Medical Center, Harvard Medical School, Boston, Massachusetts; 7 CVS Health Clinical Trial Services, Bell, Pennsylvania; 8 Division of Geriatrics and Palliative Medicine, Alpert Medical School of Brown University, Providence, Rhode Island

## Abstract

**Importance:**

Head-to-head safety comparisons of the mRNA vaccines for SARS-CoV-2 are needed for decision making; however, current evidence generalizes poorly to older adults, lacks sufficient adjustment, and inadequately captures events shortly after vaccination. Additionally, no studies to date have explored potential variation in comparative vaccine safety across subgroups with frailty or an increased risk of adverse events, information that would be useful for tailoring clinical decisions.

**Objective:**

To compare the risk of adverse events between mRNA vaccines for COVID-19 (mRNA-1273 and BNT162b2) overall, by frailty level, and by prior history of the adverse events of interest.

**Design, Setting, and Participants:**

This retrospective cohort study was conducted between December 11, 2020, and July 11, 2021, with 28 days of follow-up following the week of vaccination. A novel linked database of community pharmacy and Medicare claims data was used, representing more than 50% of the US Medicare population. Community-dwelling, fee-for-service beneficiaries aged 66 years or older who received mRNA-1273 vs BNT162b2 as their first COVID-19 vaccine were identified. Data analysis began on October 18, 2022.

**Exposure:**

Dose 1 of mRNA-1273 vs BNT162b2 vaccine.

**Main Outcomes and Measures:**

Twelve potential adverse events (eg, pulmonary embolism, thrombocytopenia purpura, and myocarditis) were assessed individually. Frailty was measured using a claims-based frailty index, with beneficiaries being categorized as nonfrail, prefrail, and frail. The risk of diagnosed COVID-19 was assessed as a secondary outcome. Generalized linear models estimated covariate-adjusted risk ratios (RRs) and risk differences (RDs) with 95% CIs.

**Results:**

This study included 6 388 196 eligible individuals who received the mRNA-1273 or BNT162b2 vaccine. Their mean (SD) age was 76.3 (7.5) years, 59.4% were women, and 86.5% were White. A total of 38.1% of individuals were categorized as prefrail and 6.0% as frail. The risk of all outcomes was low in both vaccine groups. In adjusted models, the mRNA-1273 vaccine was associated with a lower risk of pulmonary embolism (RR, 0.96 [95% CI, 0.93-1.00]; RD, 9 [95% CI, 1-16] events per 100 000 persons) and other adverse events in subgroup analyses (eg, 11.0% lower risk of thrombocytopenia purpura among individuals categorized as nonfrail). The mRNA-1273 vaccine was also associated with a lower risk of diagnosed COVID-19 (RR, 0.86 [95% CI, 0.83-0.87]), a benefit that was attenuated by frailty level (frail: RR, 0.94 [95% CI, 0.89-0.99]).

**Conclusions and Relevance:**

In this cohort study of older US adults, the mRNA-1273 vaccine was associated with a slightly lower risk of several adverse events compared with BNT162b2, possibly due to greater protection against COVID-19. Future research should seek to formally disentangle differences in vaccine safety and effectiveness and consider the role of frailty in assessments of COVID-19 vaccine performance.

## Key Points

**Question:**

Are there safety differences between mRNA vaccines for COVID-19, and do those differences vary by frailty level?

**Findings:**

In this cohort study of 6 388 196 older US adults, a 4% lower risk of pulmonary embolism, a 2% lower risk of thromboembolic events, and a 14% lower risk of diagnosed COVID-19 were observed among those who received the mRNA-1273 vaccine compared with the BNT162b2 vaccine. Although both vaccines were safe across frailty subgroups, differences were generally greater in individuals without frailty.

**Meaning:**

These findings suggest that compared with BNT162b2, mRNA-1273 was associated with a lower risk of adverse events, possibly due to improved protection against COVID-19.

## Teaser

This cohort study compares the risk of potential adverse events between mRNA vaccines mRNA-1273 and BNT162b2 for COVID-19 among older US adults.

## Introduction

As of January 2023, approximately 70% of the global population has received at least 1 COVID-19 vaccine.^1,2^ The BNT162b2 (Pfizer-BioNTech) and mRNA-1273 (Moderna) messenger RNA (mRNA) vaccines are among the most widely used,^3,4^ aligning with recommendations from public health authorities and evidence of their superior safety and efficacy relative to other products.^5,6,7^ Although the risk of serious adverse events following mRNA vaccine administration is low,^8,9^ evidence regarding their comparative safety remains limited.

Few studies have directly compared the risk of potential adverse events between mRNA vaccines, which differ in their manufacturing, administration, and immune response.^10,11,12^ Existing head-to-head comparisons of BNT162b2 and mRNA-1273 have shown small yet potentially meaningful differences in the risk of several adverse events that can vary by age and sex.^13,14^ However, current estimates generalize poorly to older adults and are derived from samples that are too small to capture rare events over a short and clinically relevant follow-up period. Further, no studies to date have assessed comparative vaccine safety within and across patient subgroups with increased frailty or history of the diagnoses identified as vaccine-associated adverse events—conditions likely to modify vaccine response and potentially contribute to differences in safety.^15,16^

Importantly, several of the potential vaccine-associated adverse events are also sequelae of SARS-CoV-2.^8,13,17,18,19^ As previously suggested,^13^ a more effective vaccine may appear to be safer for some outcomes due to the enhanced and differential prevention of COVID-19. Because of the prevalence of SARS-CoV-2 at the time of early vaccination efforts and observed differences in mRNA vaccine effectiveness,^20^ additional studies are needed to understand the extent to which differences in adverse events may be attributed to differential early effectiveness.

To inform public health recommendations and clinical decision making, we used a large population-based cohort of more than 6 million older adults to compare the risk of potential adverse events shortly after the first dose of mRNA-1273 and BNT162b2. We also assessed whether frailty and prior history of the conditions identified as potential vaccine-associated adverse events modified comparative vaccine associations.

## Methods

### Study Design and Data Sources

We conducted a retrospective cohort study using customer data from 2 large national pharmacy companies linked to Medicare claims between December 11, 2020, and July 11, 2021.^21^ We matched pharmacy customer prescription and vaccination data deterministically to the 100% Medicare enrollment files based on name, address, and date of birth. Approximately 95% of records were successfully matched, creating a cohort of more than 28 million individuals aged 65 years or older. Medicare Parts A and B were used to capture inpatient, outpatient, carrier, skilled nursing, and COVID-19 vaccine claims, and the Common Medicare Environment was used to measure sociodemographics and enrollment. The Minimum Data Set captured nursing home residence. The Brown University Institutional Review Board approved this study and waived informed consent because deidentified secondary data were used. This study followed the Strengthening the Reporting of Observational Studies in Epidemiology (STROBE) reporting guideline.

### Study Population

The study population comprised community-dwelling Medicare fee-for-service (FFS) beneficiaries aged 66 years or older who received an mRNA vaccine as their first COVID-19 vaccine dose during the study period. The study index date (ie, time 0) at which follow-up began was defined as the start (ie, Sunday) of the week that individuals received their first vaccine dose. The study population was restricted to Medicare FFS beneficiaries to capture relevant covariate information derived from FFS claims.

**Figure 1.**
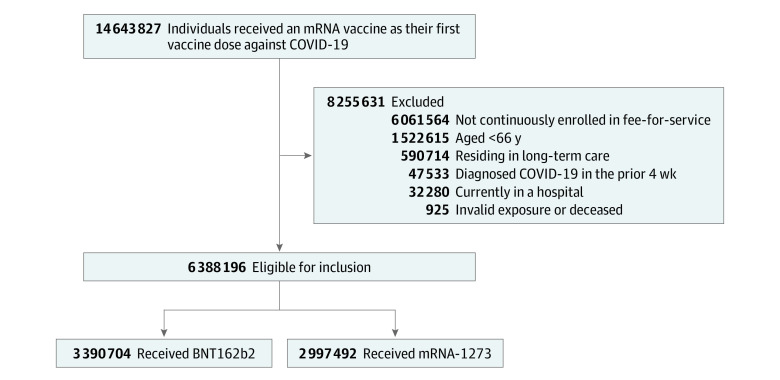
Study Flow Diagram

As of the study index date, we sequentially excluded individuals who were aged younger than 66 years, resided in long-term care, were in the hospital, were not continuously enrolled in FFS Medicare for the previous 12 months, had a documented COVID-19 diagnosis in the prior 4 weeks, had invalid vaccine data (eg, indicated as receiving both mRNA vaccines), or were deceased (Figure 1). We excluded individuals aged younger than 66 years to allow for a 1-year look-back window for covariates and to ensure continuous FFS enrollment, as Medicare eligibility begins at age 65 years for most people. Residents in long-term care were excluded due to differences in vaccination efforts in residential settings than in the community. Similar to related work,^13^ individuals with recently diagnosed COVID-19 were excluded to capture those eligible for vaccination.

### Exposure

Our primary exposure comparison of interest was the receipt of an initial dose of mRNA-1273 vs BNT162b2. Since the per-protocol and intention-to-treat estimands are identical with a single-dose exposure, we estimated both estimands in this study. The week of individuals’ first vaccine dose and vaccine manufacturer were identified using *Current Procedural Terminology* codes (0011A for mRNA-1273 and 0001A for BNT162b2) in the Medicare Part B/Carrier File and pharmacy records. To study a vaccine-naive population, we chose to assess the risk of adverse events following the first dose of an mRNA vaccine.

### Outcomes

Twelve serious adverse events identified by the US Food and Drug Administration as being potentially associated with mRNA vaccines were included as primary outcomes.^17,22^ Outcomes were measured using *International Classification of Diseases, Tenth Revision, Clinical Modification* (*ICD-10-CM*) diagnosis codes from FFS claims in Medicare Parts A and B: acute myocardial infarction, facial nerve palsy (Bell palsy), deep vein thrombosis, disseminated intravascular coagulation, encephalomyelitis or encephalitis, Guillain-Barre syndrome, hemorrhagic stroke, thrombocytopenia purpura, myocarditis or pericarditis, nonhemorrhagic stroke, pulmonary embolism, and transverse myelitis (eTable 1 in Supplement 1 presents all outcome definitions). A composite outcome that comprised events related to thromboembolic mechanisms (acute myocardial infarction, deep vein thrombosis, hemorrhagic stroke, nonhemorrhagic stroke, or pulmonary embolism) was also assessed. All *ICD-10-CM* outcome definitions were based on prior work.^17,22^

### Follow-Up

The start of follow-up for all individuals was the first day of the week (Sunday) during which the first vaccine dose was administered and continued until one of the following events: death, occurrence of an outcome (each assessed separately), or end of follow-up (28 days or July 17, 2021), whichever occurred first. Individuals with a recorded outcome on the index date who thus had 0 days of follow-up were excluded from the analysis of that outcome. We chose a 28-day follow-up period to capture adverse events most likely to be related to the vaccine.

### Baseline Covariates

As of the index date, we obtained sociodemographic characteristics (age, sex, geographic region,^23^ self-reported race and ethnicity,^24^ dual eligibility, and billing source of the vaccine claim [pharmacy, Medicare, or both sources]) for all individuals. Race and ethnicity was included as a covariate to account for potential differences in vaccine access and likelihood of vaccination and included American Indian or Alaska Native, Asian, Black, Hispanic, White, other (represents its own category derived from the Common Medicare Environment, and not the combination of several races and ethnicities), or unknown or missing. History of comorbidities within the past year was captured using FFS claims.^25^ We measured the number of weeks since an individual’s last COVID-19 diagnosis (eTable 1 in Supplement 1 presents relevant diagnosis codes), and weeks since most recent prior hospitalization, outpatient visit, and emergency department visit (individuals without these events were categorized as having no prior encounter). A claims-based frailty index was derived using a 1-year look-back window, with individuals being categorized as nonfrail (<0.15), prefrail (≥0.15 to <0.25), or frail (≥0.25).^26^ Finally, we measured zip code–level social deprivation using the American Community Survey.^27^

### Statistical Analysis

We used standardized differences to evaluate covariate balance between the mRNA-1273 and BNT162b2 vaccine groups.^28^ For the primary analysis, all individuals meeting the eligibility criteria at the index date were considered at risk for each outcome, even if they had experienced that outcome previously. Risk ratios (RRs) with 95% CIs were estimated using generalized linear models (binomial distribution and log link function). Covariates that were imbalanced (>10% standardized difference^28^) and/or determined to be clinically relevant were included in a series of models. Adjustment was conducted in stages to show the relative impact of different covariates and increasing adjustment: unadjusted (model 1); region and month of vaccination (model 2); age, sex, race and ethnicity, and frailty (model 3); and models 2 and 3 plus vaccine billing source (eg, pharmacy vs Medicare), time since prior diagnosed COVID-19, and time since prior hospitalization, outpatient visit, and emergency department visit (model 4). For outcomes with a statistically significant association in model 4, population-averaged risk differences (RDs) and 95% CIs were derived from the estimated probabilities.

#### Subgroup Analysis

We assessed potential variation in the comparative risk of adverse events across frailty level and prior history of the outcome being assessed (eTable 1 in Supplement 1). Product terms (eg, frailty × vaccine) in models 1 and 4 provided a test of effect measure modification on the multiplicative scale and the derivation of estimates within subgroups.

#### Sensitivity and Stability Analysis

First, since the vaccines were not randomly assigned and potential confounding bias was a concern, we examined the 28-day risk of hip and vertebral fractures as negative control outcomes.^29^ Second, to account for differences in dosing schedule between the vaccines, all of the primary outcomes were assessed at 21 days. Third, to contextualize the extent to which potential differences in adverse events may be related to early vaccine effectiveness, we compared the 28-day risk of diagnosed COVID-19 as a secondary outcome. For statistically significant outcomes and known sequelae of SARS-CoV-2 (eg, pulmonary embolism^19^), we used multinomial logistic regression to compare the risk of the adverse event alone, diagnosed COVID-19 alone, and the co-occurrence of the adverse event and diagnosed COVID-19.

#### Reproducibility

To provide assurance that unintentional errors in the analysis were not responsible for any findings, the cohort creation and outcome measurements were coded independently and in duplicate. Statistical analyses were conducted using SAS, version 9.4 (SAS Institute Inc), and Stata, version 17 (StataCorp LLC). Statistical significance was defined as *P* < .05. Data analysis began on October 18, 2022.

## Results

We identified 6 388 196 eligible Medicare beneficiaries who received their first dose of an mRNA vaccine; slightly over half (n = 3 390 704) received BNT162b2 (Table). Their mean (SD) age was 76.3 (7.5) years; 59.4% were women and 40.6% were men. In terms of race and ethnicity, 0.2% of individuals self-identified as American Indian or Alaska Native, 2.3% as Asian, 5.3% as Black, 0.9% as Hispanic, 86.5% as White, and 2.1% as other race or ethnicity; these data were missing or unknown for 2.7%. Diabetes (24.3%), congestive heart failure (11.9%), and cancer (15.1%) were the most common comorbidities. More than one-third of individuals were categorized as prefrail (38.1%) or frail (6.0%). Loss to follow-up due to death was very rare across all outcomes (<1.0%; eTable 2 in Supplement 1).

**Table.** Baseline Characteristics of Community-Dwelling Medicare Fee-for-Service Beneficiaries Who Received an mRNA Vaccine as Their First COVID-19 Vaccine Dose Between December 11, 2020, and July 11, 2021

Characteristic	No. of individuals (%)^a^			Standardized difference
	Overall (N = 6 388 196)	BNT162b2 (n = 3 390 704)	mRNA-1273 (n = 2 997 492)	
Age, y				
Mean (SD)	76.3 (7.5)	76.7 (7.7)	75.9 (7.2)	0.10
66-69	1 497 728 (23.5)	762 075 (22.5)	735 653 (24.5)	0.05
70-74	1 827 308 (28.6)	949 000 (28.0)	878 308 (29.3)	0.03
75-79	1 263 146 (19.8)	665 298 (19.6)	597 848 (19.9)	0.01
80-84	859 812 (13.5)	463 657 (13.7)	396 155 (13.2)	0.01
85-89	540 864 (8.5)	305 865 (9.0)	234 999 (7.8)	0.04
≥90	399 338 (6.3)	244 809 (7.2)	154 529 (5.2)	0.09
Sex				
Male	2 594 449 (40.6)	1 355 691 (40.0)	1 238 758 (41.3)	0.09
Female	3 793 747 (59.4)	2 035 013 (60.0)	1 758 734 (58.7)	0.09
Race and ethnicity				
American Indian or Alaska Native	11 119 (0.2)	5839 (0.2)	5280 (0.2)	0.00
Asian	146 800 (2.3)	81 430 (2.4)	65 370 (2.2)	0.02
Black	338 839 (5.3)	195 867 (5.8)	142 972 (4.8)	0.05
Hispanic	59 839 (0.9)	33 619 (1.0)	26 220 (0.9)	0.01
White	5 527 845 (86.5)	2 914 153 (86.0)	2 613 692 (87.2)	0.04
Other^b^	131 183 (2.1)	70 361 (2.1)	60 822 (2.0)	0.00
Unknown or missing	172 571 (2.7)	89 435 (2.6)	83 136 (2.8)	0.01
US geographic region^c^				
Northeast	1 418 946 (22.2)	764 410 (22.5)	654 536 (21.8)	0.02
Midwest	1 582 522 (24.8)	892 454 (26.3)	690 068 (23.0)	0.08
South	2 182 103 (34.2)	1 114 859 (32.9)	1 067 244 (35.6)	0.06
West	1 196 058 (18.7)	614 699 (18.1)	581 359 (19.4)	0.03
Other	8567 (0.1)	4282 (0.1)	4285 (0.1)	0.01
Social depravation index quintile				
1 (Low deprivation)	1 488 737 (23.3)	821 934 (24.2)	666 803 (22.3)	0.05
2	1 333 968 (20.9)	707 949 (20.9)	626 019 (20.9)	0.00
3	1 300 795 (20.4)	674 984 (19.9)	625 811 (20.9)	0.02
4	1 205 111 (18.9)	614 123 (18.1)	590 988 (19.7)	0.04
5 (High deprivation)	967 202 (15.1)	523 956 (15.5)	443 246 (14.8)	0.02
Missing^d^	92 383 (1.5)	47 758 (1.4)	44 625 (1.5)	0.01
Vaccine claim source^e^				
Medicare only	4 463 702 (69.9)	2 187 685 (64.5)	2 276 017 (75.9)	0.25
Pharmacy only	218 009 (3.4)	151 048 (4.5)	66 961 (2.2)	0.12
Medicare and pharmacy	1 706 485 (26.7)	1 051 971 (31.0)	654 514 (21.8)	0.21
Enrollment type				
Full dual	377 718 (5.9)	216 497 (6.4)	161 221 (5.4)	0.04
History of comorbidities in prior 6 mo				
Cancer	962 567 (15.1)	515 032 (15.2)	447 535 (14.9)	0.01
Chronic obstructive pulmonary disease	660 434 (10.3)	350 224 (10.3)	310 210 (10.4)	0.00
Congestive heart failure	762 581 (11.9)	418 635 (12.4)	343 946 (11.5)	0.03
Diabetes	1 548 953 (24.3)	821 744 (24.2)	727 209 (24.3)	0.00
Mental health conditions	701 273 (11.0)	391 377 (11.5)	309 896 (10.3)	0.04
Kidney conditions	345 915 (5.4)	194 372 (5.7)	151 543 (5.1)	0.03
Frailty in prior year (CFI score)				
Nonfrail (<0.15)	3 573 256 (55.9)	1 851 130 (54.6)	1 722 126 (57.5)	0.06
Prefrail (≥0.15 to <0.25)	2 432 991 (38.1)	1 309 473 (38.6)	1 123 518 (37.5)	0.02
Frail (≥0.25)	381 949 (6.0)	230 101 (6.8)	151 848 (5.1)	0.07
Time since last diagnosed COVID-19, wk^f^				
No documented diagnosis	6 168 746 (96.6)	3 264 827 (96.3)	2 903 919 (96.9)	0.03
1-4 (excluded from sample)	NA	NA	NA	NA
5-30	187 729 (2.9)	106 675 (3.2)	81 054 (2.7)	0.03
>30	31 721 (0.5)	19 202 (0.6)	12 519 (0.4)	0.02
Time since last hospitalization, wk^f^				
No prior hospitalization	4 124 160 (64.6)	2 150 486 (63.4)	1 973 674 (65.8)	0.05
1-4	77 488 (1.2)	43 694 (1.3)	33 794 (1.1)	0.02
5-30	469 388 (7.4)	262 581 (7.7)	206 807 (6.9)	0.03
>30	1 717 160 (26.9)	933 943 (27.5)	783 217 (26.1)	0.03
Time since last outpatient visit, wk^f^				
No prior outpatient visit	540 344 (8.5)	274 777 (8.1)	265 567 (8.9)	0.03
1-4	1 473 734 (23.1)	807 184 (23.8)	666 550 (22.2)	0.04
5-30	2 649 222 (41.5)	1 406 736 (41.5)	1 242 486 (41.5)	0.00
>30	1 724 896 (27.0)	902 007 (26.6)	822 889 (27.5)	0.02
Time since last ED visit, wk^f^				
No prior ED visit	5 017 552 (78.5)	2 641 975 (77.9)	2 375 577 (79.3)	0.03
1-4	32 042 (0.5)	17 919 (0.5)	14 123 (0.5)	0.01
5-30	207 194 (3.2)	113 872 (3.4)	93 322 (3.1)	0.01
>30	1 131 408 (17.7)	616 938 (18.2)	514 470 (17.2)	0.03

Abbreviations: CFI, claims-based frailty index; ED, emergency department; NA, not applicable.

^a^ Column percentages are reported.

^b^ Other race and ethnicity represents its own category derived from the Common Medicare Environment, and not the combination of several races and ethnicities.

^c^ Northeast comprises Connecticut, Maine, Massachusetts, New Hampshire, Rhode Island, Vermont, New Jersey, New York, and Pennsylvania. Midwest comprises Indiana, Illinois, Michigan, Ohio, Wisconsin, Iowa, Kansas, Minnesota, Missouri, Nebraska, North Dakota, and South Dakota. South comprises Delaware, Washington, DC, Florida, Georgia, Maryland, North Carolina, South Carolina, Virginia, West Virginia, Alabama, Kentucky, Mississippi, Tennessee, Arkansas, Louisiana, Oklahoma, and Texas. West comprises Arizona, Colorado, Idaho, New Mexico, Montana, Utah, Nevada, Wyoming, Alaska, California, Hawaii, Oregon, and Washington. Other comprises Puerto Rico and other US territories.

^d^ Missing represents values largely attributed to individuals with zip codes in US territories (eg, Puerto Rico), with a population size of 0, or lack of a match between data sources.

^e^ Mix of claim types represents individuals with a vaccine claim from more than 1 setting (eg, Medicare and community pharmacy).

^f^ Relative to the study index date (ie, start of the week that individuals received their first vaccine dose).

We observed few differences in baseline characteristics between groups. However, on average, individuals who received BNT162b2 were older (aged ≥90 years: 7.2% vs 5.2%; standardized difference, 0.09), were more likely to be Black (5.8% vs 4.8%; standardized difference, 0.05), and were more likely to be categorized as frail (6.8% vs 5.1%; standardized difference, 0.07).

### Comparative Risk of Adverse Events Between mRNA-1273 and BNT162b2

The risk of all adverse events was low, with each occurring in less than 1.0% of eligible individuals (eTable 3 in Supplement 1). Deep vein thrombosis and pulmonary embolism were the most frequently identified events, occurring in 0.27% and 0.23% of individuals, respectively. Disseminated intravascular coagulation (0.002%), encephalomyelitis (0.0004%), Guillain-Barre syndrome (0.0003%), and transverse myelitis (0.0002%) were very rare and were thus not examined in the adjusted and/or stratified analyses due to instability of the model estimates.

**Figure 2.**
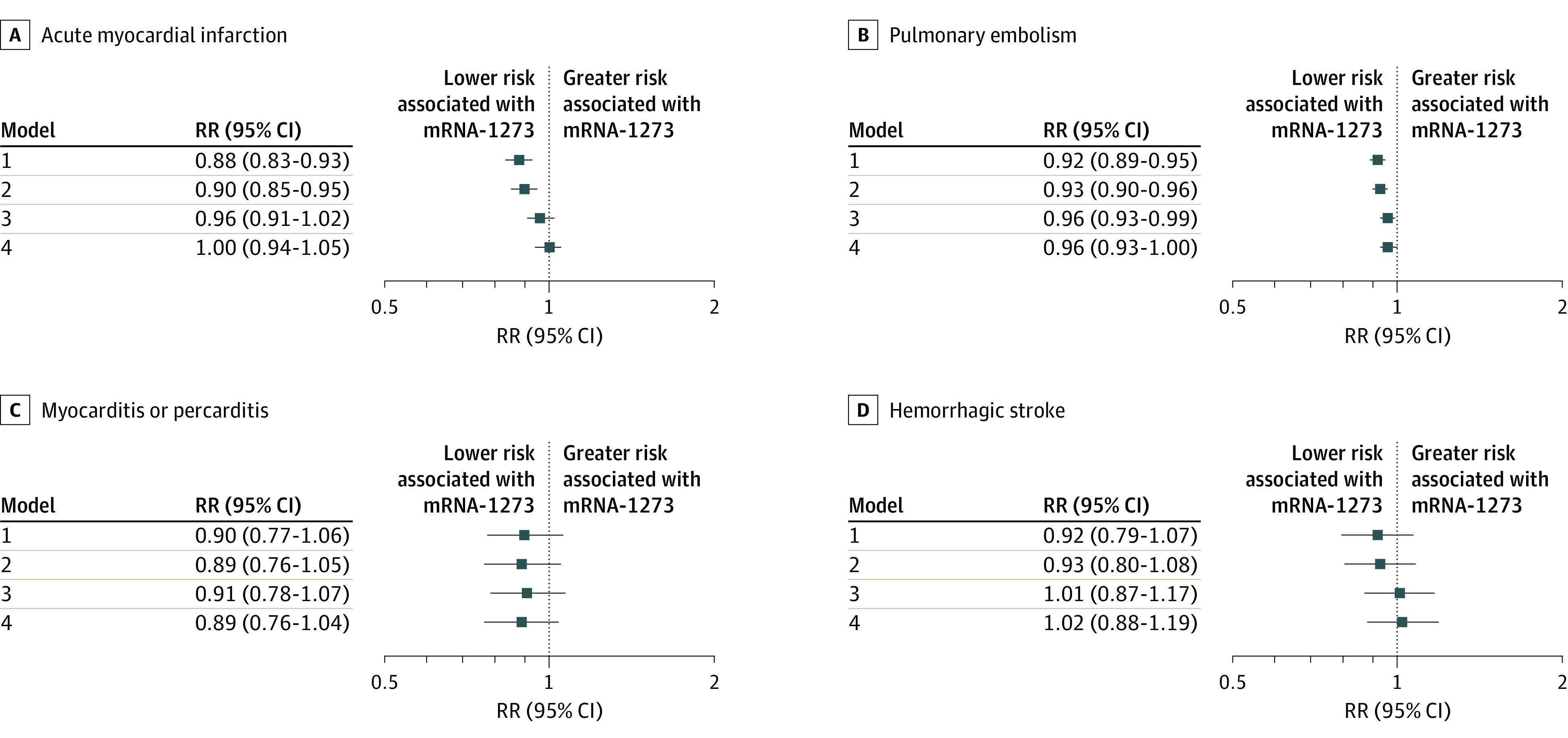
Comparative 28-Day Risk of Potential Adverse Events After First-Dose mRNA-1273 vs BNT162b2 A to D, Risk ratios (RRs) and 95% CIs for acute myocardial infarction (A), pulmonary embolism (B), myocarditis or pericarditis (C), and hemorrhagic stroke (D) were estimated using generalized linear models with a binominal distribution and log link function. Model 1 was unadjusted; model 2 was adjusted for region and month of vaccination; model 3 was adjusted for age, sex, race and ethnicity, and frailty; and model 4 was adjusted for region, month of vaccination, age, sex, race and ethnicity, frailty, claim source (eg, pharmacy and/or Medicare), time since prior documented COVID-19 infection, time since prior hospitalization, time since prior outpatient visit, and time since prior emergency department visit. Risk ratios are interpreted as the relative difference in the outcome between mRNA-1273 vs BNT162b2, whereby an RR of 1.00 represents no relative difference in risk.

Across models 1 to 4, increasing adjustment attenuated the relative differences between the mRNA vaccines (Figure 2 and eTable 4 in Supplement 1). In model 4, individuals who received mRNA-1273 had a 4.0% lower risk of pulmonary embolism and a 2.0% lower risk of the composite outcome of any thromboembolic-related event, representing 1 to 16 fewer cases of pulmonary embolism and 1 to 24 fewer thromboembolic-related adverse events per 100 000 individuals relative to BNT162b2 (pulmonary embolism: RR, 0.96 [95% CI, 0.93-1.00]; RD, 9 [95% CI, 1-16] per 100 000 individuals; composite outcome: RR, 0.98 [95% CI, 0.96-1.00]; RD, 12 [95% CI, 1-24] per 100 000 individuals). The risk of disseminated intravascular coagulation was higher among those who received mRNA-1273, but the outcome was rare and the results were not statistically significant (RR, 1.41 [95% CI, 0.95-2.10]).

### Comparative Risk of Adverse Events by Frailty Category and Prior History of the Adverse Events of Interest

**Figure 3.**
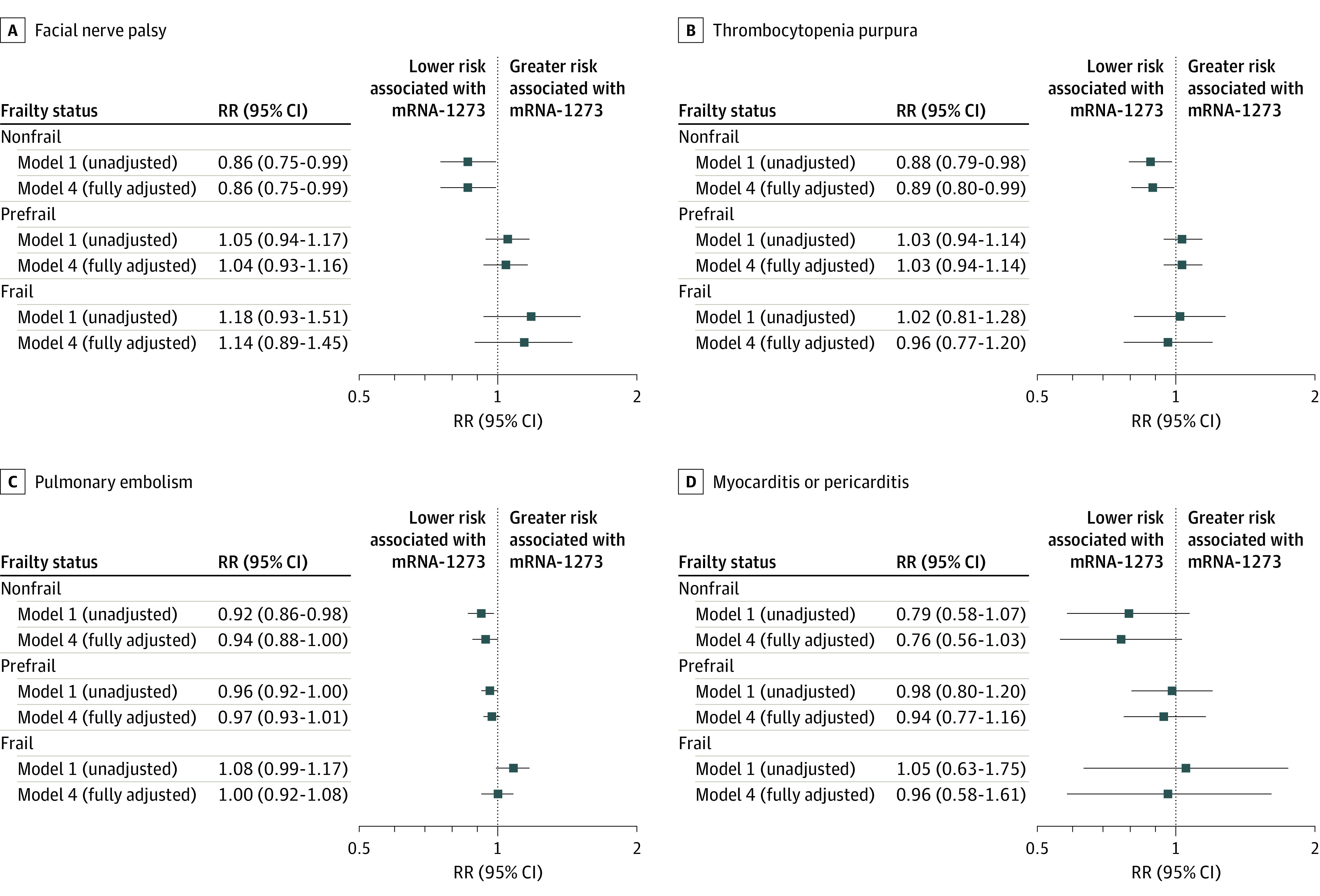
Comparative 28-Day Risk of Potential Adverse Events After First-Dose mRNA-1273 vs BNT162b2 by Frailty Level A to D, Risk ratios (RRs) and 95% CIs for facial nerve palsy (A), thrombocytopenia purpura (B), pulmonary embolism (C), and myocarditis or pericarditis (D) were estimated using generalized linear models with a binominal distribution and log link function. Interaction terms between frailty subgroup and vaccine type were included to obtain stratum-specific estimates and to formally test for modification (interaction term: *P* < .05). Model 1 was unadjusted; model 4 was adjusted for region, month of vaccination, age, sex, race and ethnicity, claim source (eg, pharmacy and/or Medicare), time since prior documented COVID-19 infection, time since prior hospitalization, time since prior outpatient visit, and time since prior emergency department visit. Risk ratios are interpreted as the relative difference in the outcome between mRNA-1273 vs BNT162b2 within each frailty subgroup, whereby an RR of 1.00 represents no relative difference in risk within that frailty subgroup. The nonfrail estimates for facial nerve palsy and prefrail estimates for thrombocytopenia purpura are the same due to rounding.

The risk of all adverse events increased with greater frailty (eTables 5 to 7 in Supplement 1). An interaction between frailty and vaccine product was observed for facial nerve palsy and thrombocytopenia purpura, with mRNA-1273 showing a 14.0% and 11.0% lower risk of both outcomes among individuals categorized as nonfrail, respectively (Figure 3 and eTables 5 to 7 in Supplement 1). A gradient across frailty was observed for several outcomes. For example, in individuals categorized as nonfrail, mRNA-1273 was associated with a 6.0% reduced risk of pulmonary embolism compared with BNT162b2 (RR, 0.94 [95% CI, 0.88-1.00]); this benefit was gradually attenuated in individuals categorized as prefrail (RR, 0.97 [95% CI, 0.93-1.01]) and frail (RR, 1.00 [95% CI, 0.92-1.08]).

The risk of each adverse event was greater among individuals who had a prior history of that condition (eTables 8 and 9 in Supplement 1). Individuals who received mRNA-1273 and had no history of deep vein thrombosis had a lower risk of incident deep vein thrombosis compared with those who received BNT162b2 (RR, 0.94 [95% CI, 0.89-1.00]; *P* = .02 for interaction).

### Sensitivity and Stability Analysis

Individuals vaccinated with mRNA-1273 had a lower risk of hip and vertebral fractures, the negative control outcomes, in the unadjusted models (RR, 0.85 [95% CI, 0.81-0.88]); however, full adjustment nullified this association (RR, 0.99 [95% CI, 0.95-1.02]), suggesting sufficient confounding control. Outcomes assessed at 21 days replicated the primary analysis (eTable 10 in Supplement 1), and the survival curves for pulmonary embolism and the composite outcome fully overlapped during the first week and began to separate at approximately day 10 and widened over time (eFigure in Supplement 1).

**Figure 4.**
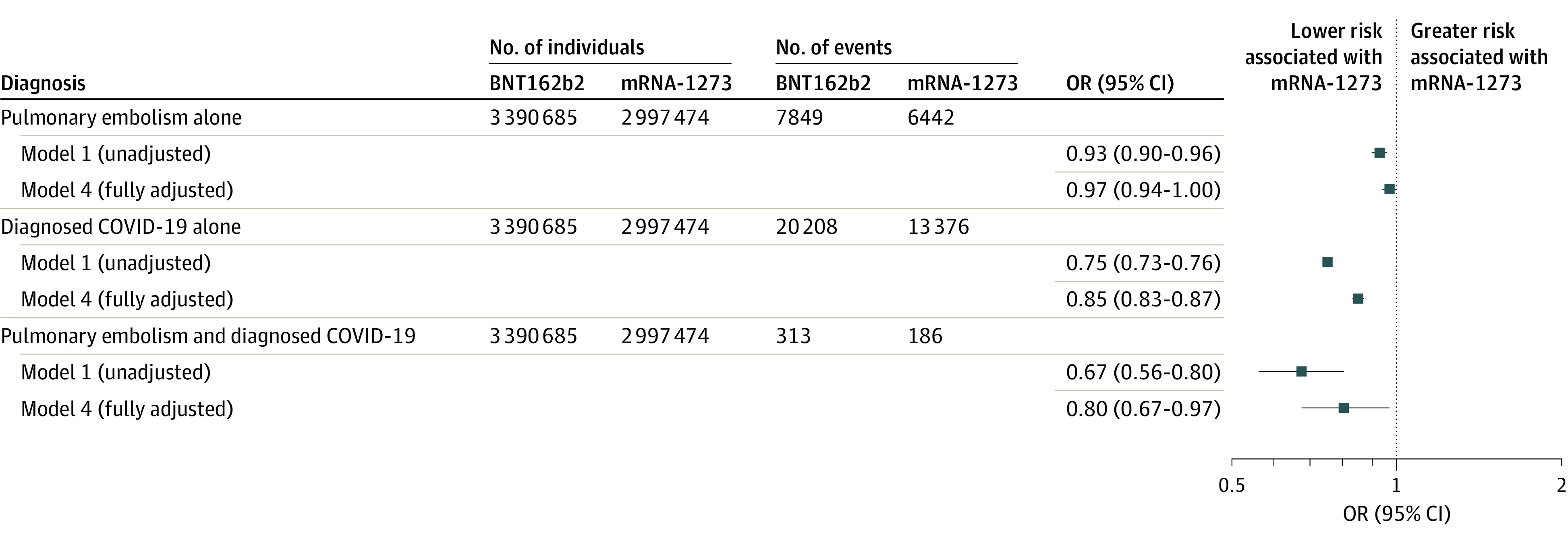
Comparative 28-Day Risk of Pulmonary Embolism, Diagnosed COVID-19, or Both Pulmonary Embolism and Diagnosed COVID-19 Odds ratios (ORs) and 95% CIs were estimated using multinomial logistic regression, comparing the risk of pulmonary embolism alone, diagnosed COVID-19 alone, or both pulmonary embolism and diagnosed COVID-19 over follow-up. Model 1 was unadjusted; model 4 was adjusted for region, month of vaccination, age, sex, race and ethnicity, claim source (eg, pharmacy and/or Medicare), time since prior documented COVID-19 infection, time since prior hospitalization, time since prior outpatient visit, and time since prior emergency department visit. Odds ratios are interpreted as the relative difference in the outcome between mRNA-1273 and BNT162b2, whereby an OR of 1.00 represents no relative difference.

The mRNA-1273 vaccine was associated with a lower risk of diagnosed COVID-19 after full adjustment (RR, 0.86 [95% CI, 0.83-0.87]); this association was attenuated in individuals categorized as frail (RR, 0.94 [95% CI, 0.89-0.99]; *P* = .01 for interaction). In a multinomial model comparing the risk of pulmonary embolism alone, diagnosed COVID-19 alone, and the co-occurrence of pulmonary embolism and diagnosed COVID-19, mRNA-1273 was associated with a significantly lower risk of COVID-19 alone (odds ratio [OR], 0.85 [95% CI, 0.83-0.87]) and the co-occurrence of pulmonary embolism and COVID-19 (OR, 0.80 [95% CI, 0.67-0.97]), but not pulmonary embolism alone (OR, 0.97 [95% CI, 0.94-1.00]; *P* = .06) (Figure 4).

## Discussion

We compared the risk of potential adverse events between the mRNA-1273 and BNT162b2 vaccines in a cohort of more than 6 million older US adults. We observed that the risk of adverse events was very low in both vaccine groups, and the vaccines did not differ in risk for most outcomes in the overall analysis. However, mRNA-1273 was associated with a lower risk of some adverse events, including pulmonary embolism, compared with BNT162b2. Notably, individuals who received mRNA-1273 also had a 14.0% lower risk of diagnosed COVID-19. Because pulmonary embolism is a sequela of COVID-19,^19^ this and potentially other observed differences in adverse events may be the result of early vaccine effectiveness and differential mitigation of COVID-19. Some variation in the comparative risk of adverse events and diagnosed COVID-19 was observed across subgroups, with mRNA-1273 showing generally larger protective associations in individuals categorized as nonfrail.

To date, a small number of studies have directly compared the safety of the BNT162b2 and mRNA-1273 vaccines and accounted for important clinical differences between groups.^13,14^ Among a cohort of US veterans, Dickerman et al^13^ compared the risk of potential vaccine-associated adverse events over 38 weeks using electronic health record data and sought to account for differential effectiveness by censoring on SARS-CoV-2. Relative to BNT162b2, mRNA-1273 was associated with a reduced risk of several outcomes, including thromboembolic events, myocarditis or pericarditis, and acute myocardial infarction. However, the authors cautioned that differences in SARS-CoV-2 incidence could not be ruled out as a potential explanation of differences in adverse events. We also observed a lower risk of several adverse events among those who received mRNA-1273 vs BNT162b2 in a larger and diverse cohort, over shorter follow-up, with robust confounding control, and across clinical subgroups. We also observed that mRNA-1273 was associated with a reduced risk of diagnosed COVID-19.^20^

Given the overlap in adverse events identified as potentially being associated with mRNA vaccines and those attributable to SARS-CoV-2, differences in safety outcomes between vaccines should be considered alongside early effectiveness.^13,19^ Differences in adverse events between vaccines may reflect the benefits of vaccination with a more effective product due to superior protection against COVID-19 and its sequelae. Results from our sensitivity analysis support the hypothesis that differences in the risk of pulmonary embolism between the vaccines are related to differential early effectiveness. Regardless of the underlying mechanism, however, the comparative reduction in morbidity associated with mRNA-1273 is notable and may have real benefits at the population level. Nonetheless, studies confirming the extent to which differences in adverse events can be attributed to early effectiveness are needed.

Assessments of potential adverse events by frailty level and their prior history of occurrence reinforced the primary analysis and provide evidence of mRNA vaccine safety in real-world and more clinically vulnerable populations. These analyses also preliminarily favor attributing the observed differences in adverse events to early effectiveness rather than safety. The mRNA-1273 vaccine was associated with generally larger reductions in adverse events and diagnosed COVID-19 among individuals categorized as nonfrail. Because frailty is known to attenuate vaccine response,^15^ the greater immunogenicity associated with mRNA-1273 may have been diminished in individuals categorized as frail, thereby reducing its degree of differential protection against COVID-19 and its sequelae.^12,30^

### Limitations

First, despite our large sample, several outcomes were too rare to examine with precision. Second, residual confounding remains a possibility and the smaller effect sizes reported herein should be interpreted with some caution. Additionally, early perceptions regarding differences in vaccine performance may have contributed to the nonrandom selection or administration of BNT162b2 and mRNA-1273. However, we adjusted for many factors and the results from the negative control outcome analysis demonstrate robustness. Third, incomplete outcome ascertainment is possible; however, with the potential exception of myocarditis,^14,18^ we do not anticipate that outcomes would be differentially captured between vaccine groups. Fourth, we cannot confirm whether the observed differences in adverse events are due to a vaccine safety signal or differential effectiveness. Fifth, our use of administrative claims without chart review makes it challenging to determine the timing of adverse events and the temporal sequencing of diagnosed COVID-19. Similarly, since our follow-up period began at the start of the week, it was possible for adverse events to occur prior to the true vaccination date; however, due to the severity of the outcomes assessed, we suspect this sequencing to be rare. Finally, we do not have data on the risks of adverse events under study in an unvaccinated comparator group.

## Conclusions

In this cohort study of older US adults, the risk of adverse events following BNT162b2 and mRNA-1273 administration was low for both mRNA vaccines, affirming their safety overall and in patient subgroups at potentially increased risk of adverse events. Because the risk of adverse events following natural infection exceeds that of either mRNA vaccine,^8,18^ vaccination with any available product should be prioritized. Nonetheless, mRNA-1273 was associated with a slightly lower risk of pulmonary embolism and other adverse events compared with BNT162b2. Because individuals who received mRNA-1273 also had a lower risk of diagnosed COVID-19, the reduced risk of adverse events in this vaccine group may represent the benefits of vaccination with a more effective product. Future research should seek to formally disentangle differences in vaccine safety and effectiveness and consider the role of frailty in assessments of COVID-19 vaccine performance.

## Notes

Supplement 1. **eTable 1.** ICD-10-CM Diagnosis Codes Used to Define the Serious Adverse Event Outcomes in This Study **eTable 2.** Participant Loss to Follow-up in the 28 Days Following the Week of the First Dose of an mRNA Vaccine Against COVID-19 (December 2020 to July 2021) **eTable 3.** Risk of Serious Adverse Events and Diagnosed COVID-19 in the 28 Days Following the Week of the First Dose of mRNA-1273 or BNT162b2 Vaccines Among Community-Dwelling Medicare Fee-for-Service Beneficiaries in the US (December 2020 to July 2021) **eTable 4.** Unadjusted and Adjusted Relative Risk of Serious Adverse Events and Diagnosed COVID-19 in the 28 Days Following the Week of the First Dose of mRNA-1273 Compared to BNT162b2 Vaccines Among Community-Dwelling Medicare Beneficiaries in the US (December 2020 to July 2021) **eTable 5.** Unadjusted and Adjusted Relative Risk of Serious Adverse Events and Diagnosed COVID-19 in the 28 Days Following the Week of the First Dose of mRNA-1273 Compared to BNT162b2 Vaccines Among Community-Dwelling Medicare Beneficiaries Categorized as Nonfrail in the US (December 2020 to July 2021) **eTable 6.** Unadjusted and Adjusted Relative Risk of Serious Adverse Events and Diagnosed COVID-19 in the 28 Days Following the Week of the First Dose of mRNA-1273 Compared to BNT162b2 Vaccines Among Community-Dwelling Medicare Beneficiaries Categorized as Prefrail in the US (December 2020 to July 2021) **eTable 7.** Unadjusted and Adjusted Relative Risk of Serious Adverse Events and Diagnosed COVID-19 in the 28 Days Following the Week of the First Dose of mRNA-1273 Compared to BNT162b2 Vaccines Among Community-Dwelling Medicare Beneficiaries Categorized as Frail in the US (December 2020 to July 2021) **eTable 8.** Unadjusted and Adjusted Relative Risk of Serious Adverse Events in the 28 Days Following the Week of the First Dose of mRNA-1273 Compared to BNT162b2 Vaccines Among Community-Dwelling Medicare Beneficiaries in the US Who Did Not Have a Recent History of the Outcome Being Assessed (December 2020 to July 2021) **eTable 9.** Unadjusted and Adjusted Relative Risk of Serious Adverse Events in the 28 Days Following the Week of the First Dose of mRNA-1273 Compared to BNT162b2 Vaccines Among Community-Dwelling Medicare Beneficiaries in the US Who Had a Recent History of the Outcome Being Assessed (December 2020 to July 2021) **eTable 10.** Unadjusted and Adjusted Relative Risk of Serious Adverse Events in the 21 Days Following the Week of the First Dose of mRNA-1273 Compared to BNT162b2 Vaccines Among Community-Dwelling Medicare Beneficiaries in the US (December 2020 to July 2021) **eFigure.** Adjusted Survival Curves for Pulmonary Embolism and Composite Outcome of Thromboembolic Events in the 28 Days Following the Week of the First Dose of mRNA-1273 Compared to BNT162b2 Vaccines Among Community-Dwelling Medicare Beneficiaries in the US (December 2020 to July 2021)

Supplement 2.
Data Sharing Statement
