# Supplementary material for: Comparative Risks of Potential Adverse Events Following COVID-19 mRNA Vaccination Among Older US Adults
Source: JAMA Netw Open. 2023 Aug 2;6(8):e2326852. doi: 10.1001/jamanetworkopen.2023.26852 (PMC10398407; doi:10.1001/jamanetworkopen.2023.26852)
Supplement: Supplement 1. — eTable 1. ICD-10-CM Diagnosis Codes Used to Define the Serious Adverse Event Outcomes in This Study eTable 2. Participant Loss to Follow-up in the 28 Days Following the Week of the First Dose of an mRNA Vaccine Against COVID-19 (December 2020 to July 2021) eTable 3. Risk of Serious Adverse Events and Diagnosed COVID-19 in the 28 Days Following the Week of the First Dose of mRNA-1273 or BNT162b2 Vaccines Among Community-Dwelling Medicare Fee-for-Service Beneficiaries in the US (December 2020 to July 2021) eTable 4. Unadjusted and Adjusted Relative Risk of Serious Adverse Events and Diagnosed COVID-19 in the 28 Days Following the Week of the First Dose of mRNA-1273 Compared to BNT162b2 Vaccines Among Community-Dwelling Medicare Beneficiaries in the US (December 2020 to July 2021) eTable 5. Unadjusted and Adjusted Relative Risk of Serious Adverse Events and Diagnosed COVID-19 in the 28 Days Following the Week of the First Dose of mRNA-1273 Compared to BNT162b2 Vaccines Among Community-Dwelling Medicare Beneficiaries Categorized as Nonfrail in the US (December 2020 to July 2021) eTable 6. Unadjusted and Adjusted Relative Risk of Serious Adverse Events and Diagnosed COVID-19 in the 28 Days Following the Week of the First Dose of mRNA-1273 Compared to BNT162b2 Vaccines Among Community-Dwelling Medicare Beneficiaries Categorized as Prefrail in the US (December 2020 to July 2021) eTable 7. Unadjusted and Adjusted Relative Risk of Serious Adverse Events and Diagnosed COVID-19 in the 28 Days Following the Week of the First Dose of mRNA-1273 Compared to BNT162b2 Vaccines Among Community-Dwelling Medicare Beneficiaries Categorized as Frail in the US (December 2020 to July 2021) eTable 8. Unadjusted and Adjusted Relative Risk of Serious Adverse Events in the 28 Days Following the Week of the First Dose of mRNA-1273 Compared to BNT162b2 Vaccines Among Community-Dwelling Medicare Beneficiaries in the US Who Did Not Have a Recent History of the Outcome Being Assessed (December 2020 [file jamanetwopen-e2326852-s001.pdf]

Supplemental Online Content

Harris DA, Hayes KN, Zullo AR, et al. Comparative risks of potential adverse events following COVID-19 mRNA vaccination among older US adults. *JAMA Netw Open*. 2023;6(8):e2326852. doi:10.1001/jamanetworkopen.2023.26852

eTable 1. ICD-10-CM Diagnosis Codes Used to Define the Serious Adverse Event Outcomes in This Study

eTable 2. Participant Loss to Follow-up in the 28 Days Following the Week of the First Dose of an mRNA Vaccine Against COVID-19 (December 2020 to July 2021)

eTable 3. Risk of Serious Adverse Events and Diagnosed COVID-19 in the 28 Days Following the Week of the First Dose of mRNA-1273 or BNT162b2 Vaccines Among Community-Dwelling Medicare Fee-for-Service Beneficiaries in the US (December 2020 to July 2021)

eTable 4. Unadjusted and Adjusted Relative Risk of Serious Adverse Events and Diagnosed COVID-19 in the 28 Days Following the Week of the First Dose of mRNA-1273 Compared to BNT162b2 Vaccines Among Community-Dwelling Medicare Beneficiaries in the US (December 2020 to July 2021)

eTable 5. Unadjusted and Adjusted Relative Risk of Serious Adverse Events and Diagnosed COVID-19 in the 28 Days Following the Week of the First Dose of mRNA-1273 Compared to BNT162b2 Vaccines Among Community-Dwelling Medicare Beneficiaries Categorized as Nonfrail in the US (December 2020 to July 2021)

eTable 6. Unadjusted and Adjusted Relative Risk of Serious Adverse Events and Diagnosed COVID-19 in the 28 Days Following the Week of the First Dose of mRNA-1273 Compared to BNT162b2 Vaccines Among Community-Dwelling Medicare Beneficiaries Categorized as Prefrail in the US (December 2020 to July 2021)

eTable 7. Unadjusted and Adjusted Relative Risk of Serious Adverse Events and Diagnosed COVID-19 in the 28 Days Following the Week of the First Dose of mRNA-1273 Compared to BNT162b2 Vaccines Among Community-Dwelling Medicare Beneficiaries Categorized as Frail in the US (December 2020 to July 2021)

eTable 8. Unadjusted and Adjusted Relative Risk of Serious Adverse Events in the 28 Days Following the Week of the First Dose of mRNA-1273 Compared to BNT162b2 Vaccines Among Community-Dwelling Medicare Beneficiaries in the US Who Did Not Have a Recent History of the Outcome Being Assessed (December 2020 to July 2021)

© 2023 Harris DA et al. *JAMA Network Open*.

eTable 9. Unadjusted and Adjusted Relative Risk of Serious Adverse Events in the 28 Days Following the Week of the First Dose of mRNA-1273 Compared to BNT162b2 Vaccines Among Community-Dwelling Medicare Beneficiaries in the US Who Had a Recent History of the Outcome Being Assessed (December 2020 to July 2021)

eTable 10. Unadjusted and Adjusted Relative Risk of Serious Adverse Events in the 21 Days Following the Week of the First Dose of mRNA-1273 Compared to BNT162b2 Vaccines Among Community-Dwelling Medicare Beneficiaries in the US (December 2020 to July 2021)

eFigure. Adjusted Survival Curves for Pulmonary Embolism and Composite Outcome of Thromboembolic Events in the 28 Days Following the Week of the First Dose of mRNA-1273 Compared to BNT162b2 Vaccines Among Community-Dwelling Medicare Beneficiaries in the US (December 2020 to July 2021)

This supplemental material has been provided by the authors to give readers additional information about their work.

eTable 1. ICD-10-CM Diagnosis Codes Used to Define the Serious Adverse Event Outcomes in This Study

Adverse Event Outcome	Criteria for event	Setting	ICD-10-CM Diagnosis Codes	Lookback window to capture prior outcome events
Acute myocardial infarction	≥ 1 diagnosis code	Inpatient	I21.01, I21.02, I21.09, I21.11, I21.19, I21.21, I21.29, I21.3, I21.4, I21.9, I21.A1, I21.A9, I22.0, I22.1, I22.2, I22.8, I22.9	365 days
Deep vein thrombosis	≥ 1 diagnosis code	Inpatient; Outpatient and professional/provider claims	I82.220, I82.3, I82.401, I82.402, I82.403, I82.409, I82.411, I82.412, I82.413, I82.419, I82.421, I82.422, I82.423, I82.429, I82.431, I82.432, I82.433, I82.439, I82.441, I82.442, I82.443, I82.449, I82.451, I82.452, I82.453, I82.459, I82.461, I82.462, I82.463, I82.469, I82.491, I82.492, I82.493<br>I82.499, I82.4Y1, I82.4Y2, I82.4Y3, I82.4Y9, I82.4Z1, I82.4Z2, I82.4Z3, I82.4Z9, I82.621, I82.622, I82.623, I82.629	365 days
Disseminated intravascular coagulation	≥ 1 diagnosis code	Inpatient; Outpatient-emergency department	D65	365 days
Encephalitis or encephalomyelitis	≥ 1 diagnosis code	Inpatient	G04.02, G04.00, G04.81, G04.90, G05.3	183 days
Facial nerve palsy (Bell's palsy)	≥ 1 diagnosis code	Inpatient; Outpatient and professional/provider claims	G51.0, G51.8, G51.9	183 days
Guillain-Barre syndrome	≥ 1 diagnosis code in the primary position	Inpatient	G61.0	365 days
Hemorrhagic stroke	≥ 1 diagnosis code	Inpatient	I61.0, I61.1, I61.2, I61.3, I61.4, I61.5, I61.6, I61.8, I61.9, I62.00, I62.01, I62.02, I62.9	365 days
Myocarditis or pericarditis	≥ 1 diagnosis code for myocarditis or pericarditis	Inpatient; Outpatient and professional/provider claims	B33.22, B33.23, I30.0, I30.1, I30.8, I30.9, I32, I41, I40.0, I40.1, I40.8, I40.9, I51.4	365 days

© 2023 Harris DA et al. *JAMA Network Open*.

Adverse Event Outcome	Criteria for event	Setting	ICD-10-CM Diagnosis Codes	Lookback window to capture prior outcome events
Non-hemorrhagic stroke	≥ 1 diagnosis code	Inpatient	I63.00, I63.011, I63.012, I63.013, I63.019 I63.02, I63.031, I63.032, I63.033, I63.039, I63.09, I63.10, I63.111, I63.112, I63.113, I63.119, I63.12, I63.131, I63.132, I63.133, I63.139, I63.19, I63.20, I63.211, I63.212, I63.213, I63.219, I63.22, I63.231, I63.232, I63.233, I63.239, I63.29, I63.30, I63.311, I63.312, I63.313, I63.319, I63.321, I63.322, I63.323, I63.329, I63.331, I63.332, I63.333, I63.339, I63.341, I63.342, I63.343, I63.349, I63.39, I63.40, I63.411, I63.412, I63.413, I63.419, I63.421, I63.422, I63.423, I63.429, I63.431, I63.432, I63.433, I63.439, I63.441, I63.442, I63.443, I63.449, I63.49, I63.50, I63.511, I63.512, I63.513, I63.519, I63.521, I63.522, I63.523, I63.529, I63.531, I63.532, I63.533, I63.539, I63.541, I63.542, I63.543, I63.549, I63.59, I63.6, I63.81, I63.89, I63.9	365 days
Pulmonary Embolism	≥ 1 diagnosis code	Inpatient; Outpatient and professional/provider claims	I26.02, I26.09, I26.92, I26.93, I26.94, I26.99	365 days
Thrombocytopenic Purpura	≥ 1 diagnosis code	Inpatient; Outpatient and professional/provider claims	D69.3	365 days
Transverse Myelitis	≥ 1 diagnosis code	Inpatient; Outpatient-emergency department	G37.3	365 days
COVID-19 diagnosis	≥ 1 diagnosis code	Inpatient; Outpatient-emergency department; Outpatient and professional/provider claims	U07.1 at any time during 2020 or 2021 OR B97.29 AND any of the following prior to April 1, 2020: J12.89, J20.8, J22, J40, J80, or J98.8	N/A
**Notes:** All outcome definitions were based on Moll K, Lufkin B, Fingar KR, et al. Background rates of adverse events of special interest for COVID-19 vaccine safety monitoring in the United States, 2019-2020. Vaccine. 2022 Nov 8:S0264-410X(22)01373-1. doi: 10.1016/j.vaccine.2022.11.003.				

© 2023 Harris DA et al. *JAMA Network Open*.

eTable 2. Participant Loss to Follow-up in the 28 Days Following the Week of the First Dose of an mRNA Vaccine Against COVID-19 (December 2020 to July 2021)

Outcome	Starting N[1]	BNT162b2			mRNA-1273		
		Total Days of Follow-Up	Average Follow-up Length (SD)	Number who censored	Total Days of Follow-Up	Average Follow-up Length (SD)	Number who censored
Acute myocardial infarction	6,388,196	94,332,467	27.82 (1.72)	6,611	83,610,969	27.89 (1.33)	3,642
Facial nerve palsy (Bell's Palsy)	6,388,185	94,339,083	27.82 (1.72)	6,889	83,615,479	27.89 (1.33)	3,837
Deep vein thrombosis	6,388,126	94,211,988	27.86 (1.92)	6,814	83,512,120	27.86 (1.56)	3,783
Disseminated intravascular coagulation	6,388,196	94,357,889	27.83 (1.69)	6,879	83,631,344	27.90 (1.29)	3,819
Encephalomyelitis	6,388,196	94,358,096	27.83 (1.69)	6,898	83,631,698	27.90 (1.24)	3,839
Guillain-Barre Syndrome	6,388,196	94,358,123	27.83 (1.69)	6,899	83,631,726	27.90 (1.29)	3,840
Hemorrhagic Stroke	6,388,196	94,354,958	27.83 (1.69)	6,817	83,628,855	27.89 (1.30)	3,779
Thrombocytopenia purpura	6,388,189	94,326,636	27.82 (1.75)	6,891	83,605,915	27.89 (1.36)	3,835
Myocarditis/pericarditis	6,388,195	94,353,553	27.83 (1.70)	6,893	83,627,942	27.90 (1.30)	3,839
Non-hemorrhagic stroke	6,388,196	94,343,458	27.82 (1.71)	6,783	83,619,476	27.90 (1.32)	3,753
Pulmonary embolism	6,388,159	94,232,299	27.79 (1.89)	6,805	83,529,008	27.87 (1.53)	3,766
Transverse myelitis	6,388,196	94,358,138	27.83 (1.69)	6,899	83,631,759	27.90 (1.29)	3,840
Thromboembolic composite[2]	6,388,097	94,065,161	27.74 (2.11)	6,339	83,392,968	27.82 (1.78)	3,447

Notes: SD=Standard deviation.
1. The total N excludes individuals who had the outcome event at time zero (i.e., zero follow-up).
2. Includes acute myocardial infarction, stroke (non-hemorrhagic and hemorrhagic), pulmonary embolism, and deep vein thrombosis.

© 2023 Harris DA et al. *JAMA Network Open*.

eTable 3. Risk of Serious Adverse Events and Diagnosed COVID-19 in the 28 Days Following the Week of the First Dose of mRNA-1273 or BNT162b2 Vaccines Among Community-Dwelling Medicare Fee-for-Service Beneficiaries in the US (December 2020 to July 2021)

Outcome	Total N[1]	Total number of events overall	Total number of events among BNT162b2	Total number of events among mRNA-1273	28-Day Risk Overall	28-Day Risk BNT162b2	28-Day Risk mRNA-1273
Acute myocardial infarction	6,388,196	4,782	2,688	2,094	0.075%	0.079%	0.070%
Facial nerve palsy (Bell's Palsy)	6,388,185	2,350	1,271	1,079	0.037%	0.037%	0.036%
Deep vein thrombosis	6,388,126	17,286	9,508	7,778	0.271%	0.280%	0.259%
Disseminated intravascular coagulation	6,388,196	103	48	55	0.002%	0.001%	0.002%
Encephalomyelitis	6,388,196	≤ 25	≤ 25	≤ 25	-	-	-
Guillain-Barre Syndrome	6,388,196	≤ 25	≤25	≤ 25	-	-	-
Hemorrhagic Stroke	6,388,196	702	387	315	0.011%	0.011%	0.011%
Thrombocytopenia purpura	6,388,189	3,389	1,846	1,543	0.053%	0.054%	0.051%
Myocarditis/pericarditis	6,388,195	611	340	271	0.010%	0.010%	0.009%
Non-hemorrhagic stroke	6,388,196	2,751	1,530	1,221	0.043%	0.045%	0.041%
Pulmonary embolism	6,388,159	14,790	8,162	6,628	0.232%	0.241%	0.221%
Transverse myelitis	6,388,196	≤ 25	≤ 25	≤ 25	-	-	-
Thromboembolic composite[2]	6,388,097	37,082	20,511	16,571	0.580%	0.605%	0.553%
COVID-19 diagnosis	6,388,196	34,084	20,522	13,562	0.533%	0.605%	0.452%

Notes:
1. The total N excludes individuals who had the outcome event at time zero (i.e., zero follow-up).
2. Includes acute myocardial infarction, stroke (non-hemorrhagic and hemorrhagic), pulmonary embolism, and deep vein thrombosis.
3. Small cells (< 25 observations) are suppressed due to privacy and data use agreements.

© 2023 Harris DA et al. *JAMA Network Open*.

eTable 4. Unadjusted and Adjusted Relative Risk of Serious Adverse Events and Diagnosed COVID-19 in the 28 Days Following the Week of the First Dose of mRNA-1273 Compared to BNT162b2 Vaccines Among Community-Dwelling Medicare Beneficiaries in the US (December 2020 to July 2021)

	**Model 1[1]**		**Model 2[2]**		**Model 3[3]**		**Model 4[4]**	
**Outcome**	**RR**	**95% CI**	**RR**	**95% CI**	**RR**	**95% CI**	**RR**	**95% CI**
Acute myocardial infarction	0.88	0.83-0.93	0.90	0.85-0.95	0.96	0.91-1.02	1.00	0.94-1.05
Bell's palsy (facial nerve palsy)	0.96	0.89-1.04	0.96	0.88-1.04	0.98	0.91-1.07	0.98	0.90-1.06
Deep vein thrombosis	0.93	0.90-0.95	0.94	0.91-0.96	0.98	0.95-1.01	0.99	0.96-1.02
Disseminated intravascular coagulation	1.30	0.88-1.91	1.30	0.88-1.92	1.44	0.97-2.12	1.41	0.95-2.10
Encephalomyelitis	0.87	0.38-1.98	-	-	-	-	-	-
Guillain-Barre Syndrome	0.68	0.25-1.87	-	-	-	-	-	-
Hemorrhagic stroke	0.92	0.79-1.07	0.93	0.80-1.08	1.01	0.87-1.17	1.02	0.88-1.19
Thrombocytopenia purpura	0.95	0.88-1.01	0.95	0.89-1.02	0.97	0.91-1.04	0.96	0.90-1.03
Myocarditis/pericarditis	0.90	0.77-1.06	0.89	0.76-1.05	0.91	0.78-1.07	0.89	0.76-1.04
Non-hemorrhagic stroke	0.90	0.84-0.97	0.92	0.85-0.99	1.00	0.93-1.08	1.02	0.94-1.10
Pulmonary embolism	0.92	0.89-0.95	0.93	0.90-0.96	0.96	0.93-0.99	**0.96**	**0.93-1.00**
Transverse myelitis	0.23	0.05-1.03	-	-	-	-	-	-
Thromboembolic composite[5]	0.91	0.90-0.93	0.93	0.91-0.95	0.97	0.95-0.99	**0.98**	**0.96-1.00**
COVID-19 diagnosis	0.75	0.73-0.76	0.76	0.74-0.78	0.80	0.78-0.81	**0.86**	**0.83-0.87**

Notes: RR=relative risk; CI=confidence interval

1. Model 1 is unadjusted; 2. Model 2 is adjusted for region and month of vaccination; 3. Model 3 is adjusted for age, sex, race, and frailty; 4. Model 4 is adjusted for age, sex, race, frailty, claim source (e.g., Medicare or community pharmacy), month of vaccination, region, and prior health services use (e.g., time since last emergency department visit), and time since prior COVID-19 infection; 5. Includes acute myocardial infarction, stroke (non-hemorrhagic and hemorrhagic), pulmonary embolism, and deep vein thrombosis; 6. Due to a small number of outcome events, lack of model convergence and/or statistical instability, not all model results are reported; 7. Statistically significant ($p < 0.05$) results from Model 4 are indicated in bold.

© 2023 Harris DA et al. *JAMA Network Open*.

eTable 5. Unadjusted and Adjusted Relative Risk of Serious Adverse Events and Diagnosed COVID-19 in the 28 Days Following the Week of the First Dose of mRNA-1273 Compared to BNT162b2 Vaccines Among Community-Dwelling Medicare Beneficiaries Categorized as Nonfrail in the US (December 2020 to July 2021)

	Non-frail (CFI < 0.15)							Ratio of the adjusted relative risks (relative, relative risks [RRR])	
				Unadjusted (Model 1)		Adjusted[1] (Model 4)			
**Outcome**	**N**	**Number of events**	**28-Day risk**	**RR**	**95% CI**	**RR**	**95% CI**	**Non-Frail RR/ Pre-Frail RR (RRR)**	**Non-Frail RR/Frail RR (RRR)**
Acute myocardial infarction	3,573,256	1,200	0.03%	0.95	0.85-1.06	1.00	0.89-1.12	0.8%	1.7%
Bell's palsy (facial nerve palsy)	3,573,253	844	0.02%	0.86	0.75-0.99	**0.86**	**0.75-0.99**	-16.8%	-24.0%
Deep vein thrombosis	3,573,244	4,387	0.12%	0.97	0.91-1.03	0.99	0.94-1.05	-0.2%	3.8%
Hemorrhagic stroke	3,573,256	208	0.01%	0.90	0.69-1.19	0.94	0.71-1.23	-14.3%	-3.9%
Thrombocytopenia purpura	3,573,254	1,424	0.04%	0.88	0.79-0.98	**0.89**	**0.80-0.99**	-13.8%	-7.2%
Myocarditis/pericarditis	3,573,256	170	0.00%	0.79	0.58-1.07	0.76	0.56-1.03	-19.6%	-21.3%
Non-hemorrhagic stroke	3,573,256	806	0.02%	0.90	0.79-1.04	0.95	0.83-1.09	-14.8%	7.8%
Pulmonary embolism	3,573,248	3,916	0.11%	0.92	0.86-0.98	0.94	0.88-1.00	-2.8%	-5.7%
Thromboembolic composite[2]	3,573,239	9,681	0.27%	0.94	0.90-0.98	0.96	0.93-1.00	-2.9%	0.5%
COVID-19 diagnosis	3,573,256	11,475	0.32%	0.83	0.80-0.86	0.85	0.82-0.88	2.4%	-9.6%

Notes: FI= frailty index; RR=relative risk; CI=confidence interval; CFI=claim's-based frailty index. Frailty was estimated using a 93-item claims-based frailty index (Kim DH, Schneeweiss S, Glynn RJ, Lipsitz LA, Rockwood K, Avorn J. Measuring Frailty in Medicare Data: Development and Validation of a Claims-Based Frailty Index. J Gerontol A Biol Sci Med Sci 2018; 73: 980–7.)

1) Models were adjusted for age, sex, race, claim source (e.g., Medicare or community pharmacy), month of vaccination, region, and prior health services use (e.g., time since last emergency department visit), and time since prior COVID-19 infection (Model 4 from the primary analysis).
2. Includes acute myocardial infarction, stroke (non-hemorrhagic and hemorrhagic), pulmonary embolism, and deep vein thrombosis.
3. Statistically significant ($p < 0.05$) results from Model 4 are indicated in bold.

© 2023 Harris DA et al. *JAMA Network Open*.

eTable 6. Unadjusted and Adjusted Relative Risk of Serious Adverse Events and Diagnosed COVID-19 in the 28 Days Following the Week of the First Dose of mRNA-1273 Compared to BNT162b2 Vaccines Among Community-Dwelling Medicare Beneficiaries Categorized as Prefrail in the US (December 2020 to July 2021)

	Pre-frail (0.15 ≤ CFI < 0.25)								
				Unadjusted (Model 1)			Adjusted[1] (Model 4)		
**Outcome**	**N**	**Number of events**	**28-Day risk**	**RR**	**95% CI**	**Interaction p-value**	**RR**	**95% CI**	**Interaction p-value**
Acute myocardial infarction	2,432,991	2,617	0.11%	0.93	0.86-1.01	0.77	0.99	0.92-1.08	0.91
Bell's palsy (facial nerve palsy)	2,432,983	1,237	0.05%	1.05	0.94-1.17	0.03	1.04	0.93-1.16	0.04
Deep vein thrombosis	2,432,954	9,679	0.40%	0.98	0.94-1.02	0.79	1.00	0.96-1.04	0.95
Hemorrhagic stroke	2,432,991	357	0.01%	1.04	0.84-1.28	0.44	1.09	0.89-1.35	0.38
Thrombocytopenia purpura	2,432,987	1,652	0.07%	1.03	0.94-1.14	0.03	1.03	0.94-1.14	0.04
Myocarditis/pericarditis	2,432,990	380	0.02%	0.98	0.80-1.20	0.24	0.94	0.77-1.16	0.24
Non-hemorrhagic stroke	2,432,991	1,398	0.06%	1.04	0.94-1.15	0.11	1.12	1.00-1.24	0.07
Pulmonary embolism	2,432,970	8,475	0.35%	0.96	0.92-1.00	0.27	0.97	0.93-1.01	0.46
Thromboembolic composite[2]	2,432,937	20,707	0.85%	0.97	0.94-1.00	0.16	0.99	0.97-1.02	0.23
COVID-19 diagnosis	2,432,991	16,049	0.66%	0.76	0.74-0.79	0.001	**0.83**	**0.81-0.86**	0.42

Notes: FI= frailty index; RR=relative risk; CI=confidence interval; CFI=claim's-based frailty index. Frailty was estimated using a 93-item claims-based frailty index (Kim DH, Schneeweiss S, Glynn RJ, Lipsitz LA, Rockwood K, Avorn J. Measuring Frailty in Medicare Data: Development and Validation of a Claims-Based Frailty Index. J Gerontol A Biol Sci Med Sci 2018; 73: 980–7.)

1. Models were adjusted for age, sex, race, claim source (e.g., Medicare or community pharmacy), month of vaccination, region, and prior health services use (e.g., time since last emergency department visit), and time since prior COVID-19 infection.
2. Includes acute myocardial infarction, stroke (non-hemorrhagic and hemorrhagic), pulmonary embolism, and deep vein thrombosis.

© 2023 Harris DA et al. *JAMA Network Open*.

eTable 7. Unadjusted and Adjusted Relative Risk of Serious Adverse Events and Diagnosed COVID-19 in the 28 Days Following the Week of the First Dose of mRNA-1273 Compared to BNT162b2 Vaccines Among Community-Dwelling Medicare Beneficiaries Categorized as Frail in the US (December 2020 to July 2021)

				Frail (CFI ≥ 0.25)					
				Unadjusted (Model 1)			Adjusted[1] (Model 4)		
**Outcome**	**N**	**Number of events**	**28-day risk**	**RR**	**95% CI**	**Interaction p-value**	**RR**	**95% CI**	**Interactio n p-value**
Acute myocardial infarction	381,949	965	0.25%	0.94	0.82-1.07	0.89	0.99	0.86-1.12	0.85
Bell's palsy (facial nerve palsy)	381,949	269	0.07%	1.18	0.93-1.51	0.03	1.14	0.89-1.45	0.05
Deep vein thrombosis	381,928	3,220	0.84%	1.00	0.93-1.07	0.51	0.96	0.89-1.03	0.42
Hemorrhagic stroke	381,949	137	0.04%	0.93	0.66-1.31	0.91	0.97	0.69-1.38	0.86
Thrombocytopenia purpura	381,948	313	0.08%	1.02	0.81-1.28	0.24	0.96	0.77-1.20	0.55
Myocarditis/pericarditi s	381,949	61	0.02%	1.05	0.63-1.75	0.34	0.96	0.58-1.61	0.43
Non-hemorrhagic stroke	381,949	547	0.14%	0.83	0.70-0.99	0.48	0.88	0.74-1.05	0.51
Pulmonary embolism	381,941	2,399	0.63%	1.08	0.99-1.17	0.002	1.00	0.92-1.08	0.26
Thromboembolic composite[2]	381,921	6,694	1.75%	0.99	0.95-1.04	0.06	0.96	0.91-1.01	0.88
COVID-19 diagnosis	381,949	6,560	1.72%	0.77	0.73-0.81	0.01	**0.94**	**0.89-0.99**	**0.01**

Notes: CFI= claims-based frailty index; RR=relative risk; CI=confidence interval; Frailty was estimated using a 93-item claims-based frailty index (Kim DH, Schneeweiss S, Glynn RJ, Lipsitz LA, Rockwood K, Avorn J. Measuring Frailty in Medicare Data: Development and Validation of a Claims-Based Frailty Index. J Gerontol A Biol Sci Med Sci 2018; 73: 980–7.)

1. Models were adjusted for age, sex, race, claim source (e.g., Medicare or community pharmacy), month of vaccination, region, and prior health services use (e.g., time since last emergency department visit), and time since prior COVID-19 infection.
2. Includes acute myocardial infarction, stroke (non-hemorrhagic and hemorrhagic), pulmonary embolism, and deep vein thrombosis.

© 2023 Harris DA et al. *JAMA Network Open*.

eTable 8. Unadjusted and Adjusted Relative Risk of Serious Adverse Events in the 28 Days Following the Week of the First Dose of mRNA-1273 Compared to BNT162b2 Vaccines Among Community-Dwelling Medicare Beneficiaries in the US Who Did Not Have a Recent History of the Outcome Being Assessed (December 2020 to July 2021)

				No recent history of the outcome[1]					
				Unadjusted (Model 1)			Adjusted[2] (Model 4)		
**Outcome**	**N**	**Number of events**	**28-Day risk**	**RR**	**95% CI**	**Interaction p-value**	**RR**	**95% CI**	**Interaction p-value**
Acute myocardial infarction	6,338,141	4,363	0.07%	0.88	0.83-0.94	0.77	0.99	0.93-1.06	0.95
Facial nerve palsy (Bell's palsy)	6,378,275	1,059	0.02%	1.03	0.91-1.16	0.47	1.04	0.92-1.17	0.52
Deep vein thrombosis	6,294,752	4,759	0.08%	0.93	0.87-0.98	0.01	**0.94**	**0.89-1.00**	**0.02**
Hemorrhagic stroke	6,382,511	675	0.01%	0.93	0.80-1.08	0.98	1.02	0.88-1.19	0.96
Thrombocytopenia purpura	6,375,171	493	0.01%	0.99	0.83-1.18	0.92	0.99	0.83-1.19	0.97
Myocarditis/pericarditis	6,382,800	339	0.01%	0.89	0.72-1.10	0.91	0.87	0.70-1.08	0.80
Non-hemorrhagic stroke	6,357,059	2,591	0.04%	0.92	0.85-0.99	0.26	1.03	0.95-1.12	0.18
Pulmonary embolism	6,320,501	3,070	0.05%	0.92	0.85-0.98	0.09	0.92	0.86-0.99	0.09

Notes: RR=relative risk; CI=confidence interval.
1. The lookback window from time zero for all outcomes was 365 days to measure prior occurrence, except for facial nerve palsy (183-day lookback).
2. Models were adjusted for age, sex, race, claim source (e.g., Medicare or community pharmacy), month of vaccination, region, and prior health services use (e.g., time since last emergency department visit), and time since prior COVID-19 infection.

© 2023 Harris DA et al. *JAMA Network Open*.

eTable 9. Unadjusted and Adjusted Relative Risk of Serious Adverse Events in the 28 Days Following the Week of the First Dose of mRNA-1273 Compared to BNT162b2 Vaccines Among Community-Dwelling Medicare Beneficiaries in the US Who Had a Recent History of the Outcome Being Assessed (December 2020 to July 2021)

				Has a recent history of the outcome[1]					
				Unadjusted (Model 1)			Adjusted[2] (Model 4)		
**Outcome**	**N**	**Number of events**	**28-Day risk**	**RR**	**95% CI**	**Interaction p-value**	**RR**	**95% CI**	**Interaction p-value**
Acute myocardial infarction	50,055	419	0.84%	0.91	0.75-1.11	0.77	0.99	0.81-1.20	0.95
Facial nerve palsy (Bell's palsy)	9,910	1,291	13.03%	0.97	0.87-1.07	0.47	0.99	0.89-1.09	0.52
Deep vein thrombosis	93,374	12,527	13.42%	1.01	0.98-1.04	0.01	**1.02**	**0.99-1.06**	**0.02**
Hemorrhagic stroke	5,685	27	0.47%	0.94	0.44-2.02	0.98	1.00	0.47-2.16	0.96
Thrombocytopenia purpura	13,018	2,896	22.25%	0.98	0.92-1.05	0.92	0.99	0.93-1.05	0.97
Myocarditis/pericarditis	5,395	272	5.04%	0.90	0.72-1.14	0.91	0.90	0.72-1.14	0.80
Non-hemorrhagic stroke	31,137	160	0.51%	0.76	0.55-1.05	0.26	0.82	0.60-1.14	0.18
Pulmonary embolism	67,658	11,720	17.32%	0.98	0.95-1.01	0.09	0.99	0.96-1.02	0.09

Notes: RR=relative risk; CI=confidence interval.
1. The lookback window from time zero for all outcomes was 365 days to measure prior occurrence, except for facial nerve palsy (183-day lookback).
2. Models were adjusted for age, sex, race, claim source (e.g., Medicare or community pharmacy), month of vaccination, region, and prior health services use (e.g., time since last emergency department visit), and time since prior COVID-19 infection.

© 2023 Harris DA et al. *JAMA Network Open*.

eTable 10. Unadjusted and Adjusted Relative Risk of Serious Adverse Events in the 21 Days Following the Week of the First Dose of mRNA-1273 Compared to BNT162b2 Vaccines Among Community-Dwelling Medicare Beneficiaries in the US (December 2020 to July 2021)

Outcome	N	Number of Events	21- Day Risk	Model 4 Adjusted RR	95% CI
Acute myocardial infarction	6,388,196	3,232	0.051%	1.02	(0.95-1.09)
Bell's palsy (facial nerve palsy)	6,388,185	1,834	0.029%	0.98	(0.89-1.08)
Deep vein thrombosis	6,388,126	13,588	0.213%	0.98	(0.95-1.01)
Disseminated intravascular coagulation	6,388,196	70	0.001%	1.58	(0.97-2.57)
Encephalomyelitis	6,388,196	≤ 25	-	-	-
Guillain-Barre Syndrome	6,388,196	≤ 25	-	-	-
Hemorrhagic stroke	6,388,196	471	0.007%	1.05	(0.87-1.26)
Thrombocytopenia purpura	6,388,189	2,795	0.044%	0.95	(0.88-1.02)
Myocarditis/pericarditis	6,388,195	458	0.007%	0.90	(0.75-1.08)
Non-hemorrhagic stroke	6,388,196	1,822	0.029%	1.02	(0.93-1.12)
Pulmonary embolism	6,388,159	11,736	0.184%	**0.96**	**(0.92-0.99)**
Transverse myelitis	6,388,196	≤ 25	-	-	-
Thromboembolic composite[5]	6,388,097	28,493	0.446%	**0.97**	**(0.95-0.99)**

Notes: RR=relative risk; CI=confidence interval
1. Model 1 is unadjusted; 2. Model 2 is adjusted for region and month of vaccination; 3. Model 3 is adjusted for age, sex, race, and frailty; 4. Model 4 is adjusted for age, sex, race, frailty, claim source (e.g., Medicare or community pharmacy), month of vaccination, region, and prior health services use (e.g., time since last emergency department visit), and time since prior COVID-19 infection; 5. Includes acute myocardial infarction, stroke (non-hemorrhagic and hemorrhagic), pulmonary embolism, and deep vein thrombosis; 6. Due to a small number of outcome events, lack of model convergence and/or statistical instability, not all model results are reported; 7. Statistically significant ($p < 0.05$) results from Model 4 are indicated in bold.

© 2023 Harris DA et al. *JAMA Network Open*.

eFigure. Adjusted Survival Curves for Pulmonary Embolism and Composite Outcome of Thromboembolic Events in the 28 Days Following the Week of the First Dose of mRNA-1273 Compared to BNT162b2 Vaccines Among Community-Dwelling Medicare Beneficiaries in the US (December 2020 to July 2021)

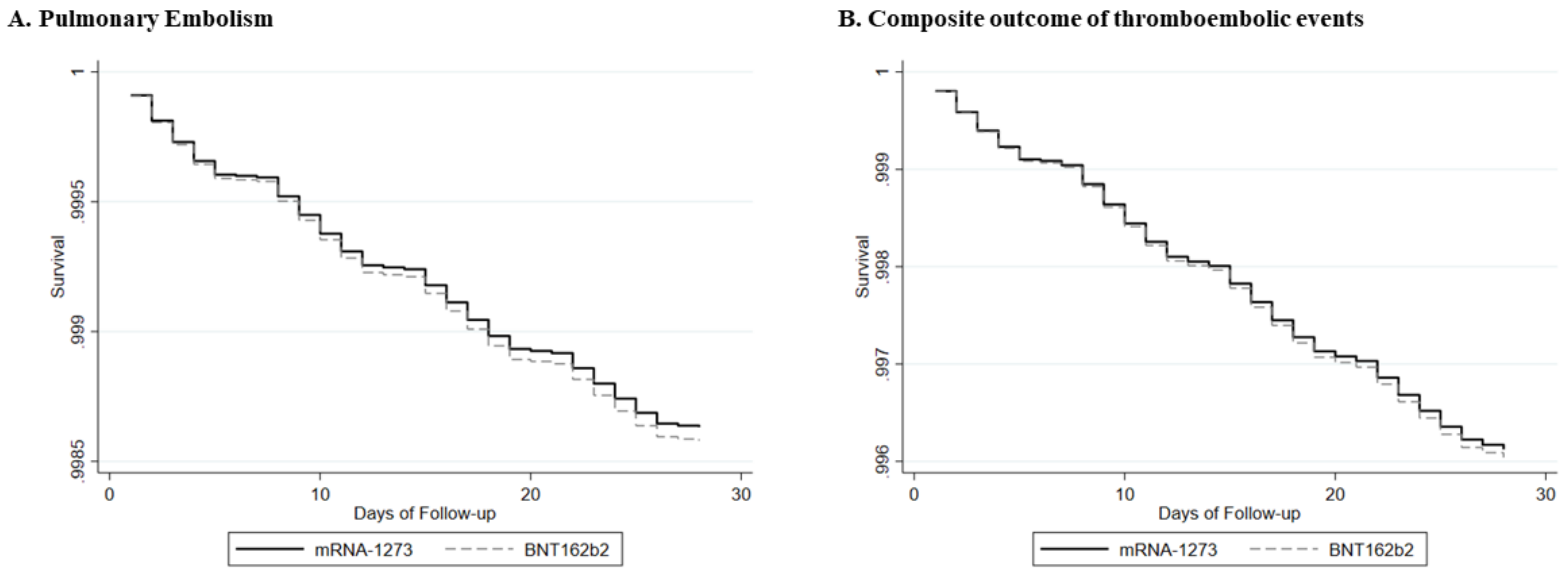

Notes: We plotted survival curves derived from Cox proportional hazards models including the Model 4 covariates to visualize and describe differences in risk over time for outcomes that were statistically significant (alpha=0.05) in the primary analysis**.** The cyclical pattern in the event dates roughly correspond to the calendar week, with fewer events being reported on weekend days (Saturday and Sunday).

© 2023 Harris DA et al. *JAMA Network Open*.
