# Supplementary material for: Comparative Risks of Potential Adverse Events Following COVID-19 mRNA Vaccination Among Older US Adults
Source: JAMA Netw Open. 2023 Aug 2;6(8):e2326852. doi: 10.1001/jamanetworkopen.2023.26852 (PMC10398407; doi:10.1001/jamanetworkopen.2023.26852)
Supplement: Supplement 2. — Data Sharing Statement [file jamanetwopen-e2326852-s002.pdf]

Data Sharing Statement

Harris. Comparative Risks of Potential Adverse Events Following COVID-19 mRNA Vaccination Among Older US Adults. *JAMA Netw Open*. Published August 02, 2023. doi:10.1001/jamanetworkopen.2023.26852

Data
Data available: No

Additional Information
Explanation for why data not available: Additional documents, such as the study protocol, analytic plan, and statistical code, can be made available to those who contact the corresponding author, Dr Daniel Harris (Daniel_harris2@brown.edu). Investigators interested in accessing the deidentified data used in this study should contact Dr Vincent Mor (vincent_mor@brown.edu) and Dr Kaleen Hayes (kaley_hayes@brown.edu) for more information about the privacy, security, and data use agreement requirements for data access.
